# Identification of structural variation in mouse genomes

**DOI:** 10.3389/fgene.2014.00192

**Published:** 2014-07-02

**Authors:** Thomas M. Keane, Kim Wong, David J. Adams, Jonathan Flint, Alexandre Reymond, Binnaz Yalcin

**Affiliations:** ^1^Wellcome Trust Sanger InstituteHinxton, Cambridge, UK; ^2^Wellcome Trust Centre for Human GeneticsOxford, UK; ^3^Center for Integrative Genomics, University of LausanneLausanne, Switzerland; ^4^Institute of Genetics and Molecular and Cellular BiologyIllkirch, France

**Keywords:** array comparative genome hybridization (aCGH), next-generation sequencing (NGS), structural variation (SV), paired-end mapping (PEM), inbred strains of mice, Heterogeneous Stock (HS), Sanger Mouse Genomes Project

## Abstract

Structural variation is variation in structure of DNA regions affecting DNA sequence length and/or orientation. It generally includes deletions, insertions, copy-number gains, inversions, and transposable elements. Traditionally, the identification of structural variation in genomes has been challenging. However, with the recent advances in high-throughput DNA sequencing and paired-end mapping (PEM) methods, the ability to identify structural variation and their respective association to human diseases has improved considerably. In this review, we describe our current knowledge of structural variation in the mouse, one of the prime model systems for studying human diseases and mammalian biology. We further present the evolutionary implications of structural variation on transposable elements. We conclude with future directions on the study of structural variation in mouse genomes that will increase our understanding of molecular architecture and functional consequences of structural variation.

## Introduction

Structural variation (SV) is generally considered as rearrangements of DNA regions affecting DNA sequence length and/or orientation in the genome of one species, and includes deletions, insertions, copy-number gains, inversions, and transposable elements. Structural variation has long been known to be pathogenic, resulting in rare genomic disorders such as well-known Charcot-Marie Tooth disease (Lupski et al., 1991; reviewed in Lupski, 1998, 2009), or more recently Koolen de Vries and 16p11.2 micro-deletion syndromes (Walters et al., 2010; Jacquemont et al., 2011; Koolen et al., 2012). Population-based SV has also begun to emerge as an important source of genomic variation contributing to common human diseases (Sebat et al., 2007; Hollox et al., 2008; Stefansson et al., 2008; Conrad et al., 2010; Pinto et al., 2010; Girirajan et al., 2011; Jarick et al., 2011; Malhotra et al., 2011; Elia et al., 2012; Helbig et al., 2014; Ramos-Quiroga et al., 2014), cancer development (Diskin et al., 2009; Stephens et al., 2011; Northcott et al., 2012; Rausch et al., 2012a; Malhotra et al., 2013; Ni et al., 2013), neuronal mosaicism in the human brain (McConnell et al., 2013) and genomic evolution (Perry et al., 2007; Itsara et al., 2010; Sudmant et al., 2013). However, the characterization of sequence flanking the breakpoints of structural variants (we call this breakpoint features), including for example micro-deletion and micro-insertion of 1 base pair (bp) up to several hundreds of bp, has remained challenging but is important with respect to not only their accurate identification, but also interpretation of their function and prediction of mechanisms by which structural variants arose (Yalcin et al., 2012a).

SVs have traditionally been observed by array comparative genome hybridization (aCGH), a method for analyzing copy number variations by measuring fluorescence between two differentially labeled DNA samples (DNA of a test sample compared to a reference sample). Using aCGH, the extent of genome-wide SV in the mouse was first demonstrated in 2007 with the detection of 80 high-confident copy number variants in 20 inbred strains of mice (Graubert et al., 2007), subsequently followed by other studies, summarized in Table 1 (Cutler et al., 2007; Akagi et al., 2008; Cahan et al., 2009; Henrichsen et al., 2009; Agam et al., 2010; Quinlan et al., 2010). These studies, however, have proven to be difficult to interpret due to their poor reproducibility (Agam et al., 2010) and inability to detect certain types of structural variants. For example inversions and insertions of novel sequence are blind to aCGH technology because inversions do not affect copy number, which is what is detected by aCGH technique, and novel sequence insertions have no copy in the reference genome.

**Table 1:** **Summary of mouse studies reporting genome-wide structural variants**.

**Technique**	**No. of SVs**	**No. of strains**	**References**
aCGH	80	20	Graubert et al., 2007
aCGH	2,094	42	Cutler et al., 2007
WGS	10,000	4	Akagi et al., 2008
aCGH	1,300	20	Cahan et al., 2009
aCGH	7,103	33^*^	Henrichsen et al., 2009
aCGH	7,196	1	Quinlan et al., 2010
aCGH	1,976	7	Agam et al., 2010
NGS	711,920	17	Yalcin et al., 2011
NGS	30,048	1	Wong et al., 2012
NGS	43	1	Simon et al., 2013

Column 1 gives the technique used in the study (aCGH, array comparative genome hybridization; WGS, whole genome sequencing; NGS, next generation sequencing). Column 2 refers to the total number of structural variants (SVs) identified and column 3, to the number of laboratory inbred mouse strains used in the study at the exception of

* that includes 21 wild-caught mice. The reference mouse strain (C57BL/6J) is excluded in the count. Column 4 is the reference to the study.

**Figure 1:**
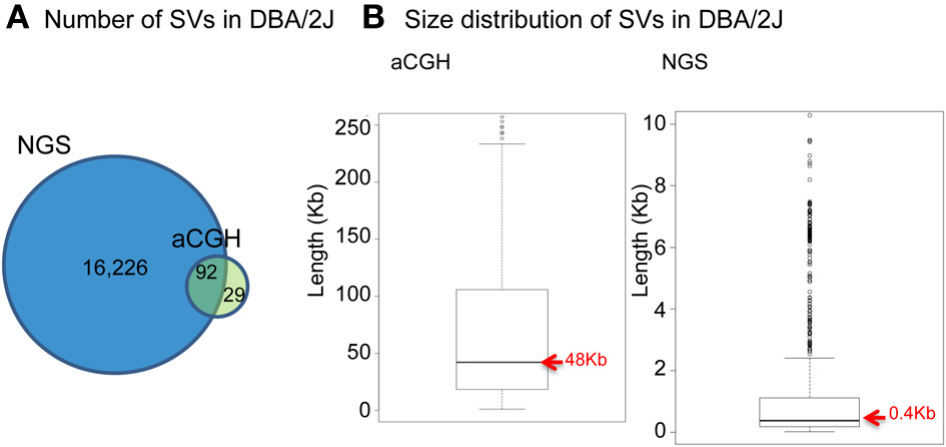
**Comparison between NGS and aCGH in inbred mouse strain DBA/2J. (A)** Venn diagram of the number of deletions detected. **(B)** Boxplot showing the size distribution of deletions.

With the emergence of next-generation sequencing (NGS) (Mardis, 2011), the Mouse Genomes Project (http://www.sanger.ac.uk/resources/mouse/genomes/) was able to sequence the entire genomes of 18 classical laboratory strains and wild-derived lines of inbred strains of mice, producing detailed maps of SV and retro-transposon elements in each mouse strain, relative to the reference mouse strain C57BL/6J (Keane et al., 2011; Nellaker et al., 2012; Wong et al., 2012; Simon et al., 2013). For the first time, this resulted in the detection of an extraordinarily larger number of structural variants than previously observed using aCGH, totaling 710,000 novel structural variants affecting 1% of the mouse genome and encompassing 10 times more total nucleotides than single nucleotide polymorphisms (Yalcin et al., 2011). As a comparison, we had identified 121 deletions in a previous aCGH study of SV in DBA/2J, with SV length ranging between minimum size of 5 kilobases (Kb) and maximum of 260 Kb (median size 48 Kb) (Agam et al., 2010), whereas in a latest NGS study of SV we found far more deletions (a total of 16,318) in that same strain, of much smaller size (minimum size of 100 bp, maximum of 10 Kb, median of 400 bp) (Figure 1).

Such genome-wide abundance in structural variation has led to several important questions: what is the molecular architecture of these variants, what are the mechanisms of SV formation and how do they impact gene function? In this review, we address these questions and redefine what we have learnt so far about the nature, origins, and role of structural variation from current studies in the mouse. Finally, we discuss the promises of novel methods which are likely to facilitate access to repeat-rich regions and assembly of complex genomic regions, in order to assess the origins and functional impact of structural variation in the most challenging regions of the mouse genome.

## Detection of structural variants using paired-end mapping methods

While most deep-sequencing applications focus on the identification of single-nucleotide polymorphisms (SNPs) or small insertion deletion polymorphisms, structural variation can also be identified from the same data. However, while the basic types of structural variants (deletions, insertions, inversions, and duplications) can be identified using a combination of computational methods, the detection of complex rearrangements remains challenging. We define complex rearrangements as those structural variants consisting of a combination of basic types that directly about each other or that are nested within each other (e.g., an inversion directly flanked by insertions, or a deletion nested within a tandem duplication).

Typically, genomic DNA of a test genome is sheared into fragments of 300–500 bp to generate a sequencing library. Short paired-reads (50–250 bp) from either extremity of the fragment (called paired-end reads) are sequenced and mapped to the reference genome. Structural variants are then called based on orientation, distance, and depth of the mapped paired-reads (also reviewed in Medvedev et al., 2009; Alkan et al., 2011). Depending on the size and type of structural variant, these methods exploit read pairs (Korbel et al., 2007; Chen et al., 2009), split-reads (Ye et al., 2009; Albers et al., 2011), single end clusters and read depth (Simpson et al., 2010).

**Figure 2:**
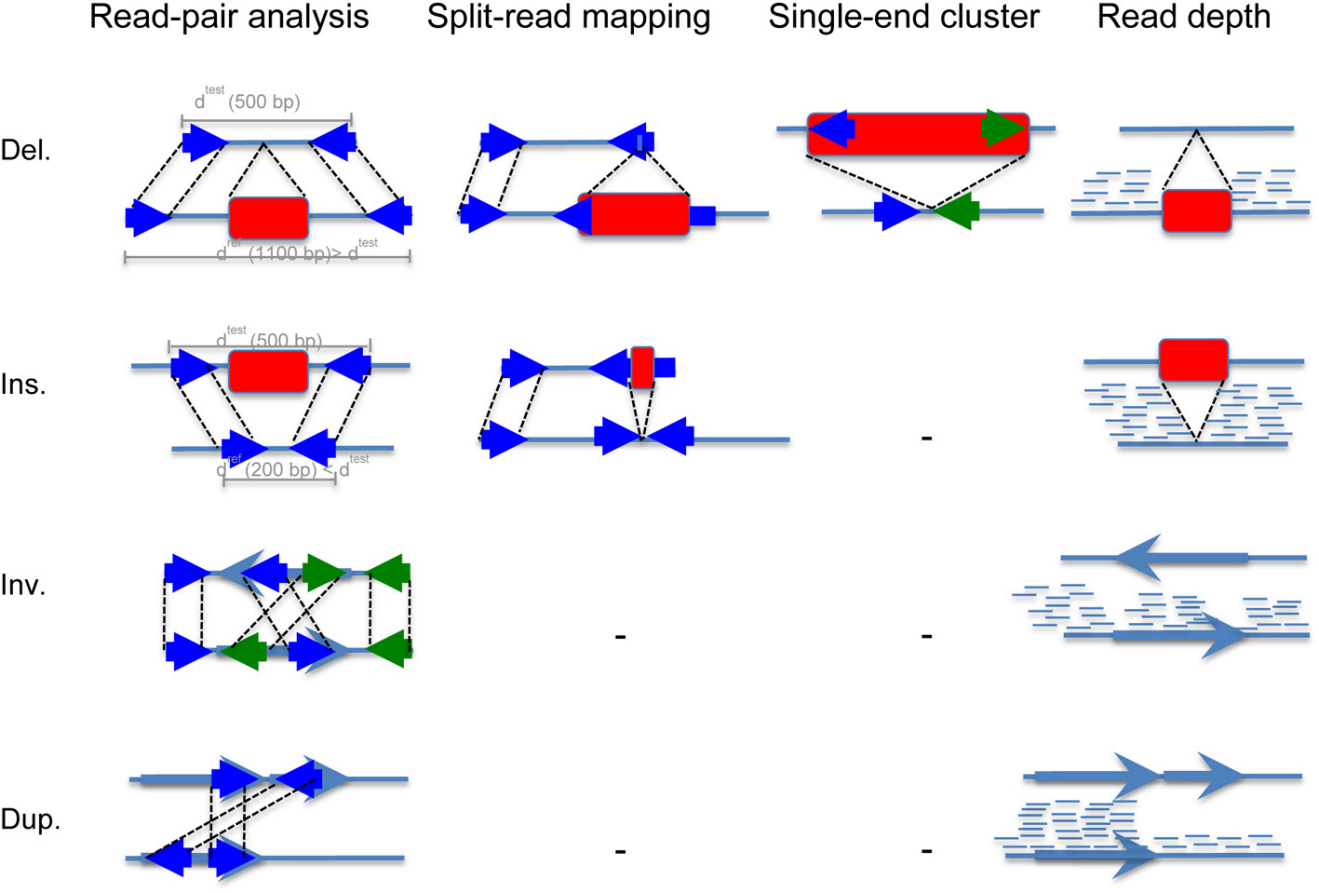
**Read mapping patterns used by computational methods to detect basic structural variation from NGS data**. This figure shows the principle of SV identification using (i) read-pair analysis, (ii) split-read mapping, (iii) single end cluster analysis, and (iv) read depth analysis. Deletions and insertions are represented using red rectangles, and inversions and duplications using light blue arrows. Reads are represented using solid dark blue arrows. The first step consists in sequencing a test genome. Typically, the genomic test DNA is fragmented into chunks of 300–500 bp. Then, reads of 50–250 bp are sequenced from either side of each fragment (we call these paired-end reads). The second step consists in mapping these paired-end reads to the mouse reference genome. A rightward facing arrow denotes a positive strand alignment, and leftward a negative strand alignment. (i) In the read-pair analysis approach, when the paired-end reads are mapping in the correct orientation (“+/−” is normal) but to a distance that is significantly larger than the average fragment length. If we suppose this distance to be 1100 bp, it suggests a deletion of 600 bp, whereas if the distance is smaller than the fragment length, for example 200 bp, it suggests an insertion of 300 bp. When the two sequenced ends of two fragments map back to the reference genome in the wrong orientation (“+/+” and “−/−”), and at a distance that is significantly larger than the size of the fragment itself, this indicates an inversion. Finally, when paired-end reads map with orientation “−/+” to a large distance, it suggest tandem duplication. (ii) In the split-read approach, one of the paired-end reads map to the reference genome while its mate contains the structural variant, typically a deletion or an insertion of small length. (iii) In the single-end cluster analysis, one of the paired-end reads maps to the reference while its mate map to the inserted sequence that can be either *de novo* sequence or repeat element such as LINE, SINE, or ERV. (iv) Finally, the read depth approach takes advantage of the high coverage of next generation sequencing that makes it possible to detect copy number changes. Of note, the coverage drops at insertion and inversion breakpoints, which when combined with paired-end reads analysis makes the SV call highly reliable.

The most widely used methods are read pair and read depth methods. Read pair based methods analyze distance and orientation of paired reads to infer deletion, insertion, inversion and tandem duplication events as shown in Figure 2. When the paired-end reads are mapping in the correct orientation (“+/−” is normal) but to a distance that is significantly larger than the average fragment length, this suggests a deletion, whereas if the distance is smaller than the fragment length, it suggests an insertion. When the two sequenced ends map back to the reference genome in the wrong orientation (“+/+” and “−/−”), and at a distance that is significantly larger than the size of the fragment itself, this indicates an inversion. Finally, when paired-end reads map with orientation “−/+” to a large distance, it suggest tandem duplication. In the single-end cluster analysis, one of the paired-end reads maps to the reference while its mate map to the inserted sequence (*de novo* sequence or repeat element insertion). Read depth methods take advantage of the high coverage of next generation sequencing to infer increase or decrease of reads at a locus. When the coverage is higher than the expected genome coverage, duplication is inferred, whereas when it is smaller or null, deletion is inferred. Once the structural variant is detected using these analyses, breakpoint refinement is typically achieved using local sequence assembly.

Remarkably, in the past several years many algorithms have been developed to discover basic structural variation in paired-end next generation sequencing data. There are over 50 programs to date (Table 2), however none is as yet considered to reach a community standard and only a handful combine multiple methods for the detection of structural variation (Medvedev et al., 2010; Wong et al., 2010; Rausch et al., 2012b; Sindi et al., 2012; Hart et al., 2013). Accurate structural variant calling depends on many factors such as sequencing library biases, read length, uniform sequencing coverage, and proximity of SVs to repeat sequences. Some of the most frequent sequencing library biases that can detrimentally affect SV detection are high PCR duplicates, non-normal fragment size distributions, and uneven representation of the genome at varying levels of GC content. Therefore, false negative rates of most studies remain high (20–30%) compared to SNP calling (<5%). False positive rates are also high and are often caused by misalignment of the short reads and sometimes by reference genome assembly errors.

**Table 2:** **Algorithms for the detection of structural variation**.

**Algorithm**	**Description**	**Download**	**References**
BreakDancer	Predicts del, ins, inv, and translocations using PEM. Performance examined in an ind. with acute myeloid leukemia and samples from the 1000 Genomes trio. Compared with VariationHunter and MoDIL	http://gmt.genome.wustl.edu/breakdancer/current/	Chen et al., 2009
CNAseg	Identifies CNVs from NGS data. Uses depth of coverage to estimate copy number states in cancer and normal samples	http://www.compbio.group.cam.ac.uk/software.html	Ivakhno et al., 2010
cnD	HMM that uses read coverage to determine genomic copy number. Tested on short read sequence data generated from re-sequencing chr. 17 of the mouse strains A/J and CAST/EiJ with the Illumina platform	http://www.sanger.ac.uk/resources/software/cnd.html	Simpson et al., 2010
cn.MOPS	Mixture Of PoissonS Bayesian approach to detect CNVs. Compared with mrFast, EWT, JointSLM, CNV-Seq, and FREEC using data from a male HapMap individual and high coverage data from the 1000 Genomes Project	http://www.bioinf.jku.at/software/cnmops	Klambauer et al., 2012
CNVer	Method that supplements the depth-of-coverage with PEM information, where mate pairs mapping discordantly to the reference serve to indicate the presence of variation	http://compbio.cs.toronto.edu/cnver	Medvedev et al., 2010
CNVnator	Method for CNV discovery and genotyping from read-depth analysis of personal genome sequencing	http://sv.gersteinlab.org/cnvnator	Abyzov et al., 2011
CNV-Seq	Method to detect CNV using shotgun sequencing	http://tiger.dbs.nus.edu.sg/CNV-seq	Xie and Tammi, 2009
CREST	Clipping Reveals Structure, uses NGS reads with partial alignments to a ref. to map SVs at nucleotide level resolution. Used for 5 pediatric acute lymphoblastic leukemias and a human melanoma cell line	http://www.stjuderesearch.org/site/lab/zhang	Wang et al., 2011
DELLY	Integrates paired-end and split-read analysis	www.korbel.embl.de/software.html	Rausch et al., 2012b
Dindel	Bayesian method to call small indels by realigning reads to candidate haplotypes that represent alternative sequence to the reference, using a split-read approach. Used in the 1000 Genomes Project call sets	http://www.sanger.ac.uk/resources/software/dindel	Albers et al., 2011
EWT	Event-wise testing, method based on significance testing. Error rate tested using the analysis of chromosome 1 from paired-end shotgun sequence data (30×) on 5 individuals	http://rdxplorer.sourceforge.net	Yoon et al., 2009
FREEC	Control-FREE Copy number caller that automatically normalizes and segments copy number profiles	http://bioinfo-out.curie.fr/projects/freec	Boeva et al., 2011
GASV-PRO	Combines both paired read and read depth signals into a probabilistic model for greater specificity	http://compbio.cs.brown.edu/software	Sindi et al., 2012
GenomeSTRiP	Genome STRucture In Populations, toolkit for discovering and genotyping structural variations using sequencing data. Twenty to thirty genomes required to get good results	http://www.broadinstitute.org/software/genomestrip/download-genome-strip	Handsaker et al., 2011
HYDRA	Localizes SV breakpoints by PEM. Uses a similar clustering strategy to VariationHunter. Accuracy evaluated using WGS slit-read mappings. Maps repetitive elements such as transposons and SD	http://code.google.com/p/hydra-sv	Quinlan et al., 2010
inGAP-sv	Scheme that uses abnormally mapped read pairs. Possible to distinguish HOM and HET variants. Compared with VariationHunter, Breakdancer, PEMer, Spanner, Cortex, and Pindel	http://ingap.sourceforge.net	Qi and Zhao, 2011
JointSLM	Allows to detect common CNVs among individuals using depth of coverage	http://www.mybiosoftware.com/population-genetics/11185	Magi et al., 2011
MoDIL	Detection of small indels from clone-end sequencing with mixtures of distributions	http://compbio.cs.toronto.edu/modil	Lee et al., 2009
mrFast	Allows for the prediction of absolute copy-number variation of duplicated segments and genes	http://mrfast.sourceforge.net	Alkan et al., 2009
PEMer	Compatible with several NGS platforms. Simulation-based error models, yielding confidence-values for each SV	http://sv.gersteinlab.org/pemer	Korbel et al., 2009
Pindel	A pattern growth approach, to detect breakpoints of large deletions and medium-sized insertions from PEM reads	http://www.ebi.ac.uk	Ye et al., 2009
RetroSeq	Detects non-reference mobile elements such as LINE, SINE, and ERV. Accuracy evaluated using a trio from the 1000 Genomes Project	https://github.com/tk2/RetroSeq	Keane et al., 2013
SoftSearch	Combines three analyses: split-read, read-pair, and single-end cluster. Tested using low coverage HapMap samples and high-coverage 122 gene dataset. Performance compared with SVSeq2, DELLY, BrakDancer, and CREST	http://bioinformaticstools.mayo.edu	Hart et al., 2013
SPANNER	SV detection for the pilot phase of the 1000 Genomes Project using low-coverage WGS of 179 ind. from 4 pop., high-coverage seq. of 2 mother-father-child trios, and exon targeted seq. of 697 ind. from 7 pop	https://github.com/chipstewart/Spanner	Abecasis et al., 2010
SplazerS	Method for split-read mapping, where a read may be interrupted by a gap in the read-to-reference alignment	http://www.seqan.de/projects	Emde et al., 2012
Splitread	Detects SV and indels from 1 bp to 1 Mb in exome data sets. Uses one end-anchored placements to cluster the mappings of subsequences of unanchored ends to identify size, content, and location	http://splitread.sourceforge.net	Karakoc et al., 2012
SRiC	Split-read identification, calibrated (SRiC). Validated using a representative data from the 1000 Genomes Project		Zhang et al., 2011b
SVDetect	Identify discordant mate-pairs derived from NGS data produced by the Illumina GA and ABI SOLiD platforms	http://svdetect.sourceforge.net	Zeitouni et al., 2010
SVMerge	Pipeline integrating several existing callers followed by *de novo* assembly. Applied to the analysis of a HapMap trio	http://svmerge.sourceforge.net	Wong et al., 2010
SVSeq2	Split-read mapping for low-coverage sequence data	http://www.engr.uconn.edu/~jiz08001	Zhang et al., 2012
VariationHunter	Gives combinatorial formulations for the SV detection between a reference genome sequence and a NG-based, paired-end, whole genome shotgun-sequenced individual	http://compbio.cs.sfu.ca/strvar.htm	Hormozdiari et al., 2009

Column 1 names the algorithm (alphabetical order); column 2 gives a description of the method and its application; column 3 cites the URL for software download and column 4 is the reference to the study. Note that de novo assembly algorithms are not listed in this table. PEM, Paired-End Mapping; CNVs, Copy Number Variants; NGS, Next-Generation Sequencing; SVs, Structural Variants; SD, Segmental Duplication; WGS, Whole Genome Sequencing; pop., population; ind., individual; ref., reference; seq., sequencing; ins, insertion; del, deletion; inv, inversion.

There is a growing awareness of complex structural variants (Berger et al., 2011; Stephens et al., 2011; Quinlan and Hall, 2012; Yalcin et al., 2012a; Malhotra et al., 2013), however, their genome-wide detection is much more challenging and less intuitive as they often generate ambiguous paired-end mapping patterns. Complex structural variants are very often completely or partially missed, or incorrectly classified because a single method on its own might not be sufficient to capture the whole complexity of the structural variant (e.g., an apparent deletion and inversion may be simultaneously part of a tandem duplication region). Thus, it is important to combine multiple methods, something that the community has begun to do. Sindi and colleagues, for example, used an algorithm combining both read pairs and read depth signals into a probabilistic model implemented in a software GASV-PRO that significantly improves detection specificity (Sindi et al., 2012). Rausch and colleagues have developed DELLY that integrates short insert paired-ends, long-range mate-pairs and split-read alignments to accurately delineate genomic rearrangements at single-nucleotide resolution (Rausch et al., 2012b). In our studies, we used SVMerge (Wong et al., 2010), a pipeline that integrates structural variation calls from five existing software, and validates breakpoints using local *de novo* assembly.

**Figure 3:**
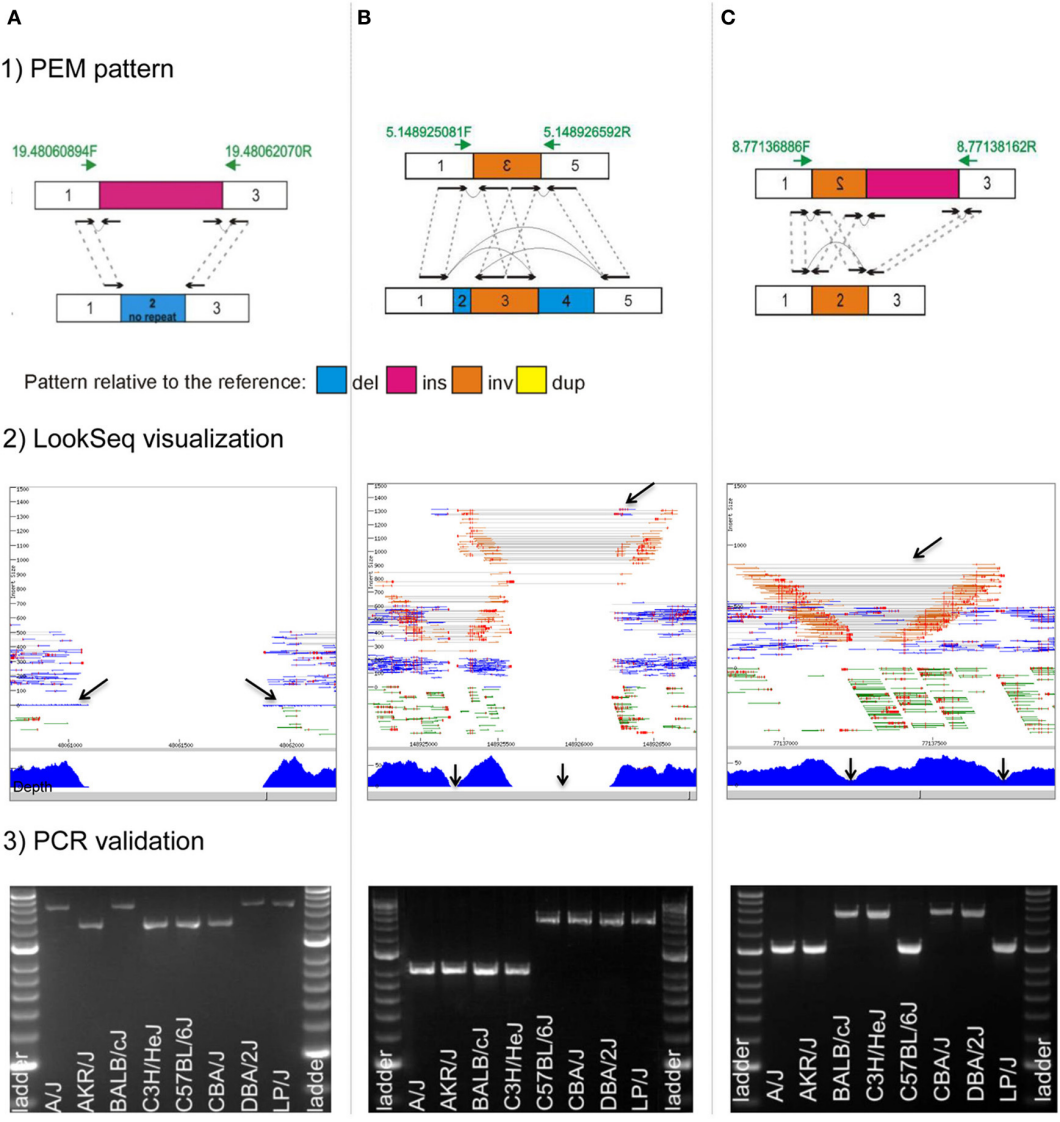
**Complex rearrangements in mouse genomes**. We highlight three examples of complex rearrangements that cause ambiguous signals during their detection (for a full list of complex rearrangements see Yalcin et al., 2012a): **(A)**, a deletion directly flanked by an insertion; **(B)**, an inversion directly flanked by two deletions; and **(C)**, an inversion directly flanked by an insertion. For each complex rearrangements, we provide: (1) a drawing of the paired-end mapping (PEM) pattern, (2) an illustration using the short read visualization tool LookSeq (Manske and Kwiatkowski, 2009), and (3) PCR validation. We draw paired-end reads (black arrows) and how they map to the reference genome (dashed gray lines). Green arrows represent primer pairs used for PCR validation. PCR amplification was carried out across eight inbred strains of mice (A/J, AKR/J, BALB/cJ, C3H/HeJ, C57BL/6J, CBA/J, DBA/2J, and LP/J), which are the parental strains of the Heterogenesous Stock population (Valdar et al., 2006). Hyperladder II is the size marker. Genomic coordinates refer to the mm9 mouse assembly. **(A)** Deletion of 836 bp directly flanked by an insertion of 1200 bp on mouse chromosome 19 (chr19: 48,061,057–48,061,892 bp) in mouse strains A/J, BALB/cJ, DBA/2J, and LP/J. In LookSeq, the two back arrows show singleton reads suggesting an insertion (their mates are within the inserted sequence). Read depth is null but paired-end reads in support of the deletion are missing because of the insertion. PCR in four strains (A/J, BALB/cJ, DBA/2J, and LP/J) does not show directly the presence of the 836-bp deletion but instead reveals the presence of an insertion of about 400 bp that is in fact the size difference between the deletion and the insertion. **(B)** Inversion of 325 bp on mouse chromosome 5 (chr5: 148,925,249–148,925,573 bp), directly flanked on the left by a deletion of 71 bp (chr5: 148,925,178–148,925,248 bp) and on the right by another deletion of 645 bp (chr5: 148,925,574–148,926,218 bp). In LookSeq, the top arrow shows the PEM pattern of the deletion. Normally, the underlying read depth should be null, however, it is only null at the regions shown by the two bottom arrows. This is caused by an intervening inversion. PCR in four strains (A/J, AKR/J, BALB/cJ, and C3H/HeJ) confirms the presence of the two deletions. **(C)** An inversion of 548 bp on mouse chromosome 8 (chr8: 77,137,213–77,137,760 bp) directly flanked by an insertion of 400 bp in mouse strain BALB/cJ, C3H/HeJ, CBA/J, and DBA/2J. In LookSeq, the bottom arrows show a dip in the coverage; on the right, it is caused by an insertion and on the left by an inversion. The presence of the insertion results in missing reads (“−/−”), supporting the inversion. PCR shows an amplification band of about 1400 bp in BALB/cJ, C3H/HeJ, CBA/J, and DBA/2J, whereas, in the remaining strains, the band is at about 1000 bp. This confirms the insertion of 400 bp in BALB/cJ, C3H/HeJ, CBA/J, and DBA/2J.

Unbiased exploration of next-generation sequencing data is laborious, however it is essential for deciphering the true complex nature of structural variants. Toward this goal, we visualized read mappings to the whole of mouse chromosome 19 as well as a random set of regions on other chromosomes using the short-read visualization tool LookSeq (Manske and Kwiatkowski, 2009) in 17 inbred strains of laboratory mice (Yalcin et al., 2012a) as well as in C57BL/6J mice (Simon et al., 2013). We were able to recognize classical paired-end mapping (PEM) patterns, but unexpectedly we were also able to detect a number of other patterns, of greater diversity and complexity that would have been missed or miscalled by existing computational SV detection methods. When two (or more) structural variants co-localize at a locus in the genome (right next to each other), or when one or more structural variants are embedded within another one of larger size (nested), it creates confusing paired-end mapping patterns and incoherent read depth. Figure 3 highlights some complex rearrangements that cause conflicting signals during automatic detection. For example, a deletion directly flanked by a large insertion is characterized by null read depth as expected, however paired reads supporting the deletion are missing because of the insertion. However, we showed that it is possible to train genome-wide computational analysis to detect most of these atypical patterns using integration of multiple detection methods (Wong et al., 2010).

In conclusion, to study the whole diversity and complexity of structural variants, future algorithms need to integrate multiple signals and sequence analyses features based on what we have learnt so far about the architecture of structural variants, while visual approaches will continue to increase our understanding of complex forms of structural variants such as inversions and translocations that remain to be fully resolved. It is important to gain better sensitivity and specificity in the identification of structural variants especially those that have complex architecture to study accurately their impact on diseases such as tumor heterogeneity (Russnes et al., 2011), and on the evolution of genomes.

## Functional impact of structural variants

The functional impact of structural variants is still controversial in the literature. On one hand, some studies showed that SNPs are more likely to contribute to individual phenotypic differences than structural variants (Conrad et al., 2010; Keane et al., 2011); on the other hand, several studies have estimated the impact of structural variation using its effect on gene expression, and these estimates ranged from 10 to 74% (Stranger et al., 2007; Cahan et al., 2009; Henrichsen et al., 2009; Yalcin et al., 2011). It has also been reported that structural variation can influence gene expression both spatially and temporally (Chaignat et al., 2011), including genes outside of SV margins (Henrichsen et al., 2009), and can do so through chromatin conformation changes (Gheldof et al., 2013). The influence of structural variation on gene expression is specifically reviewed in Harewood et al. (2012).

Interpreting the phenotypic consequences of structural variation can be done using different methods. In this review, we describe three methods with specific emphasis on genome wide association studies. Genome wide association studies (GWASs) identify genomic loci associated with individual differences (these regions are called Quantitative Trait Loci, QTLs) using large populations of outbred mice, while taking advantage of recombinants that have naturally accumulated during breeding (Valdar et al., 2006; Yalcin et al., 2010). When combined with the availability of full genome sequences, GWASs in outbred mice are providing significant advances into the understanding of the genotype-phenotype relationship (reviewed in Yalcin and Flint, 2012), especially the impact of structural variants on phenotypic differences.

To test causality of a structural variant within a QTL region, Richard Mott and colleagues have developed a statistical test (called merge) to identify genomic variants likely to be functional from those less likely to be functional (Yalcin et al., 2005). Unexpectedly, very few SVs (only 12) out of about 100,000 SVs present in classical inbred strains of mice (Yalcin et al., 2011) overlapped with a gene within QTL regions identified using an outbred population of mice known as the Heterogenous Stock mice (Talbot et al., 1999; Valdar et al., 2006; Yalcin et al., 2011). Table 3 lists these structural variants associated with quantitative traits in outbred mice. These were amongst the larger effect size QTLs. Although the number of SVs causing phenotypic differences is small, it is expected that these SVs will provide significant insights into gene function. We highlight two examples in the next paragraph.

**Table 3:** **Structural variants associated with quantitative traits in outbred mice**.

**Chr**	**Start**	**Stop**	**Type**	**Gene**	**Region**	**Quantitative trait**
1	175158884	175158885	Ins	*Fcer1a*	Upstream	Mean platelet volume
2	144402760	144402971	SINE Ins	*Sec23b*	Intron	OFT total activity
4	49690362	49690363	Del	*Grin3a*	Intron	HP cellular proliferation marker
4	108951263	108951264	IAP Ins	*Eps15*	Upstream	Home cage activity
4	130038388	130038389	SINE Ins	*Snrnp40*	Intron	T-cells: %CD3
7	90731819	90731820	IAP Ins	*Tmc3*	Upstream	Wound healing
7	111397607	111479433	Ins	*Trim5*	Exon	Mean cellular hemoglobin
7	111504989	111505193	Del	*Trim30b*	UTR	Mean cellular hemoglobin
8	87957244	87957245	LINE Ins	*4921524J17Rik*	Upstream	Mean cellular volume
11	115106127	115106250	Del	*Tmem104*	UTR	Serum urea concentration
13	113783196	113783359	Del	*Gm6320*	Upstream	HP cellular proliferation marker
17	34483681	34483682	Del	*H2-Ea*	Upstream	T-cells: CD4/CD8 ratio

Columns 1, 2, and 3 give positional information about the structural variant (coordinates refer to the mm9 mouse assembly). Column 4 is the type of the variant. Column 5 and 6 give information about the underlying gene. Column 7 is the quantitative trait associated with the structural variant. Ins, insertion; Del, deletion; UTR, untranslated region; SINE, short interspersed nuclear element; LINE, long interspersed nuclear element; IAP, intracisternal A-particle; HP, Hippocampus; OFT, open field test.

**Figure 4:**
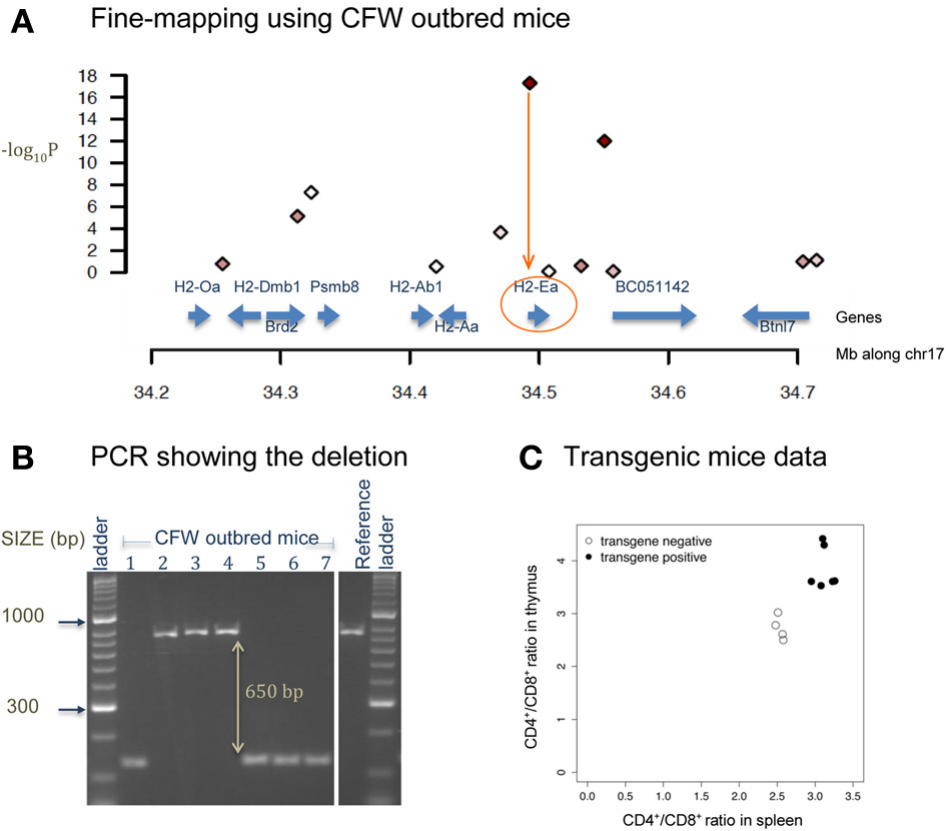
**Deletion in *H2-Ea* affects CD4^+^/CD8^+^ ratio. (A)** The x-axis is the position along mouse chromosome 17 (Mb). The y-axis shows the significance level of the association between CD4^+^/CD8^+^ ratio and a set of bi-allelic markers (represented using polygons) using a population of 200 commercially available outbred mice (CFW mice Yalcin et al., 2010). Markers with strong association (−log10P > 10) are colored in red. Strongest association is within the promoter region of *H2-Ea*. **(B)** PCR image of *H2-Ea* reveals a 600-bp deletion in 8 CFW mice. **(C)** Plot of mouse transgenic data with and without the deletion. The x-axis is the CD4^+^/CD8^+^ ratio in the spleen and the y-axis in the thymus. White circles are measures from transgene negative mice so with no deletion. Black circles are measures from transgene positive mice (with the deletion). Apart from the deletion, the genetic background of these mice is identical.

**Figure 5:**
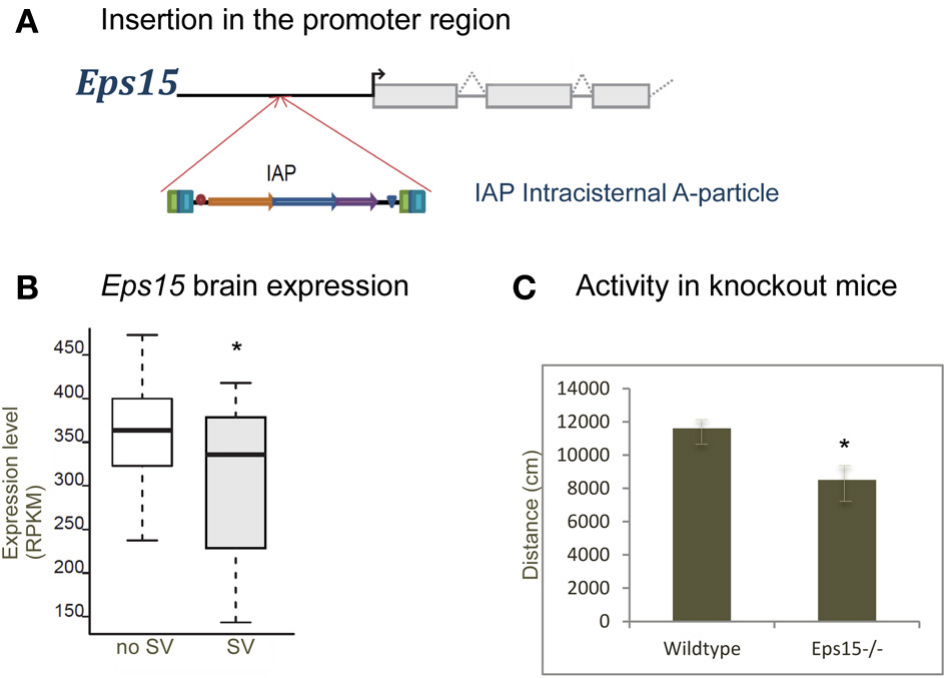
**Insertion in *Eps15* modulates activity. (A)** A transposable element (Intracisternal A-particle) of 6400 bp has inserted in the promoter region of *Eps15*. **(B)** Boxplot showing expression in the brain measured using RNA-Seq in mice with and without the structural variant (RPKM, reads per kilobase per million mapped reads). There is a decrease of expression with the presence of the insertion. **(C)** Data from the mouse knockout of *Eps15*, showing a significant decrease of home cage activity compared to matched wildtype mice (^*^p-value < 0.05).

Figure 4 shows a deletion of 600 bp lying within the promoter region of *H2-Ea* (histocompatibility 2, class II antigen E alpha) that is affecting CD4^+^/CD8^+^ ratio in T lymphocytes. This locus was fine-mapped to single-gene resolution using a population of commercial outbred mice (CFW) (Yalcin et al., 2010). Causality was confirmed using mouse transgenic data with and without the deletion. The ratio of CD4^+^/CD8^+^ was significantly increased in transgene positive mice with the deletion when compared to transgene negative mice (without the deletion), both in the spleen and in the thymus. Figure 5 illustrates a transposable element, an intracisternal A-particle (IAP) element of 6400 bp, which has inserted in the promoter region of *Eps15* (Epidermal Growth Factor Receptor Pathway Substrate 15). This variant modulates home cage activity in outbred mice. There is a decrease of expression in the brain in mice with the IAP element. Data from the mouse knockout of *Eps15* also show a significant decrease of home cage activity when compared to matched wildtype mice.

A second way to assess the phenotypic consequences of structural variation is to undertake a comprehensive phenotypic comparison between two closely related sub-strains of mice, and examine the relationship between structural variants and phenotypic changes between these strains. In a recent study, comparing phenotypic and genomic analysis of C57BL/6J and C57BL/6N sub-strains, 15 structural variants differentiating C57BL/6J and C57BL/6N were identified encompassing genic regions (Table 4). It includes three structural variants that have MP (Mammalian Phenotype) terms that coincide with the phenotype differentiating C57BL/6J and C57BL/6N. The first is an intronic LINE insertion found in the intron of *Chl1* (Cell adhesion molecule with homology to L1CAM). C57BL/6N mice displayed abnormal spatial memory in the Morris water maze test compared to C57BL/6J mice. Interestingly, knockout mice of *Chl1* also show abnormal spatial working memory. The second is an intronic ERV insertion in *Rptor* (Regulatory associated protein of MTOR, complex 1) in C57BL/6J mice. These mice were characterized by decreased fat mass and blood glucose. Knockout mice of *Rptor* interestingly also showed decreased fat mass and blood glucose amongst other metabolic phenotypes. The third is the well-known deletion at the *Nnt* (Nicotinamide nucleotide transhydrogenase) locus (Freeman et al., 2006) in C57BL/6J, which is associated with significantly impaired glucose tolerance.

**Table 4:** **Structural variants differentiating C57BL/6J and C57BL/6N**.

**Chr**	**Start**	**Stop**	**Type**	**Gene**	**Region**
2	70619835	70620080	SINE Ins	Tlk1	Intron
3	60336036	60336037	Del (large)	Mbnl1	Intron
4	101954274	101954395	Del	Pde4b	Intron
4	116051393	116051799	MaLR Ins	Mast2	Intron
6	103669536	103676487	LINE Ins	Chl1	Intron
7	92095990	92096149	Del	Vmn2r65	Exon
7	27636128	27748456	Ins	Cyp2a22	Entire
7	139306094	139307981	MaLR Ins	Cpxm2	Intron
8	16716381	16716382	Del (large)	Csmd1	Intron
9	58544415	58546304	MaLR Ins	2410076I21Rik	Intron
10	32536420	32543464	LINE Ins	Nkain2	Intron
11	119560391	119566827	MTA Ins	Rptor	Intron
12	42023964	42032747	Del	Immp2l	Intron
13	120164268	120164269	Del (large)	Nnt	Intron
19	12863187	12863188	Del (1800 bp)	Zfp91	Intron

Columns 1, 2, and 3 give positional information about the structural variant (coordinates refer to the mm9 mouse assembly). Column 4 is the type of the variant. Column 5 and 6 give information about the underlying gene. Ins, insertion; Del, deletion; LINE, Long Interspersed Nuclear Element; IAP, Intracisternal A-particle; SINE, Short Interspersed Nuclear Element; MaLR, Mammalian-Apparent Long-Terminal Repeat Retrotransposon; MTA, Mammalian Transposable Element; VNTR, Variable Number Tandem Repeat.

A third way is to search for structural variants that affect a coding region of a gene, potentially creating a null or hypomorphic allele. We found about 50 structural variants encompassing a coding segment (Yalcin et al., 2011; reviewed in Yalcin et al., 2012b), affecting eleven already known genes (*Amd2*, *Defb8*, *Fv1*, *Skint4*, *Skint3*, *Skint9*, *Soat1*, *Tas2r103*, *Tas2r120*, *Trim5*, and *Trim12a*) (Best et al., 1996; Persson et al., 1999; Bauer et al., 2001; Nelson et al., 2005; Boyden et al., 2008; Tareen et al., 2009; Wu et al., 2010) and, in some cases, are giving rise to specific phenotype in mice. For example, a deletion of 1342 bp affecting the fourth coding exon of *Fv1* (Friend-virus-susceptibility-1) is associated with retrovirus replication (Best et al., 1996; Yalcin et al., 2011), and a deletion of 6817 bp on the first exon of Soat1 (Sterol O-acyltransferase 1) results in hair interior defects (Wu et al., 2010; Yalcin et al., 2011).

Human GWAS have shown that common SNPs (minor allele frequency >5%) explain only some fraction of the heritability, suggesting that SVs might also be contributing to individual phenotypic variation (Manolio et al., 2009). Results presented in this review suggest that, given the abundance of structural variants in mouse genomes, SVs make less of a contribution to individual phenotypic variation than SNPs. However, when they do, structural variants have a large effect size on the phenotype, providing a unique opportunity to investigate the relationship between structural variants and phenotypic differences, at a molecular as well as mechanistic level.

## Evolutionary implications and transposable elements

Transposable elements (TEs) have been highly influential in shaping the structure and evolution of mammalian genomes, as exemplified by TE-derived sequence contributing between 38 and 69% of genomic sequence (Buzdin, 2004; Cordaux and Batzer, 2009; Shapiro, 2010; de Koning et al., 2011). TE insertions also can influence the transcription, translation or function of genes. Functional effects of TE insertions include their regulation of transcription by acting as alternative promoters or as enhancer elements and via the generation of antisense transcripts, or of transcriptional silencers. TEs are classified on the basis of their transposition mechanism (Goodier and Kazazian, 2008). Class I retrotransposon propagates in the host genome through an intermediate RNA step, requiring a reverse transcriptase to revert it to DNA before insertion into the genome. Class II DNA transposons do not have an RNA intermediate, and translocate with the aid of transposases and DNA polymerase. The overwhelming majority, over 96%, of TEs in the mouse genome, are of the retrotransposon type. These are further classified into three distinct classes: short interspersed nuclear elements (SINEs), long interspersed nuclear elements (LINEs), and the endogenous retrovirus (ERV) superfamily (Stocking and Kozak, 2008). The ERV elements are ancient remnants of exogenous virus infections, consisting of internal sequence that encodes viral genes that are flanked by long terminal repeats (LTRs). Therefore, TEs provide a potential source of variants detrimental to the host by altering pre-existing gene function.

Previous studies examined two ERV families in eight mouse strains (IAP or ETn/MusD elements in C57BL/6J, A/J, DBA/2J, SPRET/EiJ, CAST/EiJ, MOLF/EiJ, WSB/EiJ, and 129X1/SvJ) (van de Lagemaat et al., 2006; Quinlan et al., 2010; Li et al., 2012), with one study in particular focusing on intronic insertions (Zhang et al., 2011a) and another exploring LINE variation in four strains (A/J, DBA/2J, 129S1/SvImJ, and 129X1/SvJ) (Akagi et al., 2008). However, the largest genome-wide survey of TE polymorphism in multiple laboratory mouse strains was carried out as part of the Mouse Genomes Project (Yalcin et al., 2011; Nellaker et al., 2012). There were two types of polymorphic TE to be cataloged; those that are present in the reference genome and not present in one or more other strain; and those that are not present in the reference genome and present in one or more other strain. In total, 103,798 TE variants (TEVs) (28,951 SINEs, 40,074 LINEs, and 34,773 ERVs) were computationally predicted among the 17 sequenced mouse strains in addition to the C57BL/6J reference strain. By placing the TE insertions within a primary phylogeny, it was possible to observe the relative expansions of all the TE families over an approximate 2 million years time period. This primary phylogeny matched the phylogeny expected from the heritage of the mouse strains (Beck et al., 2000). This analysis revealed the historic expansion of ERV families, most notably IAPs, in laboratory strains. Another interesting family are the MuLV family which arose recently and thus is found in a smaller number of copies that together show a higher fraction of variable elements.

TEV density varies by chromosome, by local nucleotide composition (G + C content) (Filipski et al., 1973; Macaya et al., 1976; Thiery et al., 1976), and by position relative to functional sequence, such as exons. LINE TEVs show a bias for being located in A + T-rich sequence, whilst SINE TEVs tend to reside in G + C-rich sequence (Korenberg and Rykowski, 1988; Boyle et al., 1990). It was also observed that ERV TEVs are more heterogeneous than SINEs or LINEs in their G + C bias, with MuLV TEVs being as enriched in high G + C sequence as SINEs. Interestingly, by contrast to monomorphic TEs, polymorphic TEVs are more unevenly distributed among the chromosomes (having accounted for G + C content) with, for example, chromosome 19 exhibiting a significant enrichment of SINEs and the X chromosome showing a strong deficit of all three TEV classes (Nellaker et al., 2012). The depletion of polymorphic LINEs on the X chromosome was previously seen in a study of four mouse strains (A/J, DBA/2J, 129S1/SvImJ, and 129X1/SvJ) (Akagi et al., 2008). TEVs from all three classes show strong and significant depletions in protein-coding gene exons, implying that such insertions are strongly deleterious (assuming that most TEVs across the noncoding genome are neutral or deleterious). The significant deficits of ERV or LINE TEVs in introns indicate that many were deleterious and thus were selectively purged over these strains' evolutionary history. These observations agree with previous findings that LINE TE insertions are less tolerated within gene-rich sequence (Kvikstad and Makova, 2010).

A strong orientation bias is evident for each of the three TE classes (32.6, 41.7, and 41.6% for ERV, LINE, and SINE TEVs, respectively) (Nellaker et al., 2012). The orientation bias for IAP TEVs was recently reported to be 25.9% for a redundant set of 3317 intronic IAPs (Li et al., 2012). The strong biases for ERVs and, to a lesser extent for LINEs, are consistent with these elements being depleted from introns. The large set of TEVs examined in the genome-wide analysis allowed the authors to infer whether the location of a TEV within a gene structure affects the strength by which it is purified from the population. Orientation bias was significantly stronger for ERV TEVs within middle or last introns, and for SINE TEVs within first introns (Nellaker et al., 2012). A recent study of 161 mouse ERV TEVs identified their strongest intronic orientation bias to be in the close vicinity of exon boundaries (Zhang et al., 2011a).

Indeed, using a stringent statistical re-sampling approach to take into account confounding influences of strain and expression divergence, TEVs were found to be twice as likely to reside in a differentially expressed gene as expected by chance (Nellaker et al., 2012). However, when TEVs are considered with other forms of potential co-segregating mutations (SNPs, indels, and other structural variations), only 34 TEVs passed a stringent genome-wide test, and these TEVs contain significantly fewer LINEs than the null expectation that all TEV classes have equal effects (Nellaker et al., 2012). While it has been extensively documented in the literature that *de novo* LINE insertions can cause changes in gene expression, it appears that, in *Mus musculus*, purifying selection has preferentially purged such variants. However, given that the proportion of expression heritability attributable to TEVs generally is no more than 10% (Yalcin et al., 2011).

To summarize, transposable elements make up almost half of the mouse genome (Gogvadze and Buzdin, 2009) and importantly their activity is the most prevalent mechanism for generating large structural variations in laboratory inbred mouse strains (Yalcin et al., 2011). However, as we demonstrated in this review, transposable elements appear to be under strong purifying selection for deleterious insertions with the majority of insertions observable in present day mouse strains having little phenotypic effects (Nellaker et al., 2012).

## Data access and visualization

The entire set of structural variation calls across 18 mouse genomes (129P2/OlaHsd, 129S1/SvImJ, 129S5SvEv^Brd^, A/J, AKR/J, BALB/cJ, C3H/HeJ, C57BL/6NJ, CAST/EiJ, CBA/J, DBA/2J, FVB/NJ, LP/J, NOD/ShiLtJ, NZO/HILtJ, PWK/PhJ, SPRET/EiJ, and WSB/EiJ) have been posted on the following ftp site ftp://ftp-mouse.sanger.ac.uk/. Data sets described in this review are also available under accession numbers “estd118” (Yalcin et al., 2011), “estd185” (Yalcin et al., 2012a), “estd200” (Wong et al., 2012), and “estd204” (Simon et al., 2013) from the Database of Genomic Variants Archive (DGVa).

**Figure 6:**
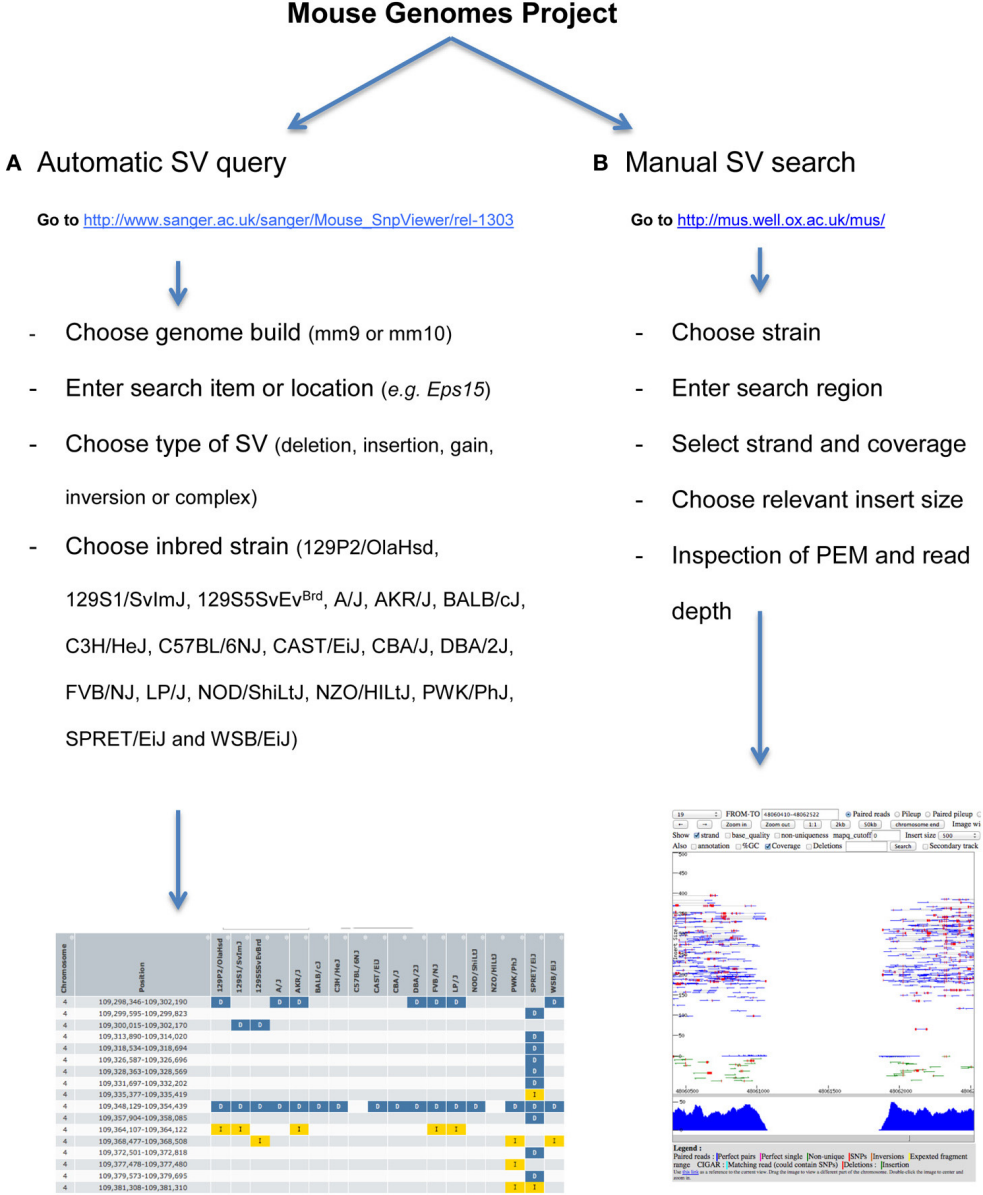
**How to access and query the data automatically and manually. (A)** Workflow of how to automatically query structural variants. Our work was published relative to mm9 Genome Build, but data can also be visualized directly onto mm10. A gene name or genomic region can be searched for simple and complex structural variants. Results can be exported as TSV and CSV format. **(B)** Workflow of how to manually search for structural variants. To do this, we use LookSeq (Manske and Kwiatkowski, 2009) as a Web-based tool to visualize paired end reads NGS data. The choice of the insert size depends on the size of the underlying structural variant, so that when the variant is large the insert size should also be large. Types of structural variants can be recognized using our comprehensive catalog of paired end mapping (PEM) patterns described in Yalcin et al. (2012a).

The project website (http://www.sanger.ac.uk/resources/mouse/genomes/) provides tools to automatically search for structural variants by location, gene, strain, type, and functional impact. A workflow of the procedure is explained in Figure 6A. Results can be exported as TSV and CSV format. Specificity and sensitivity of automatic SV calls are described in detail in Yalcin et al. (2011). To access and query the data manually, visualization of alignments (both at base-pair and read-pair levels) can be done using LookSeq (Figure 6B) (Manske and Kwiatkowski, 2009), a Web-based tool to visualize paired end reads NGS data or using the Integrative Genomics Viewer (IGV) (Robinson et al., 2011; Thorvaldsdottir et al., 2013). Structural variants can be visually identified using our comprehensive catalog of paired end mapping (PEM) patterns described in Yalcin et al. (2012a).

## Future work and concluding remarks

The current approaches for cataloging mutations are primarily based on aligning sequencing reads to the appropriate reference genome to identify SNPs, indels, and structural variations. The majority of SV discovery methods to date have been based on observing patterns of clusters of aberrant read mappings to the reference genome. However, for many groups of strains or individuals there are many haplotypes that are not present on the reference genome and therefore are excluded from the catalog of mutations. This is especially true for the wild-derived mouse strains such as SPRET/EiJ, CAST/EiJ, and PWK/PhJ. So while the current approaches can often detect the presence of a non-reference haplotype in the form of a large insertion, they are blind to sequence variation occurring on the haplotype.

One solution to this problem is to create data structures capable of representing all of the haplotypes present in a group of related samples. In a recent study, Iqbal et al. developed de Bruijn graph methods for detecting and genotyping simple and complex genetic variants in an individual or population without a reference genome and were able to discover more than 3 Mb of sequence absent from the human reference genome (Iqbal et al., 2012).

The String Graph Assembler (SGA) was the first sequence assembly pipeline for next-generation data based on sequence overlaps (Simpson and Durbin, 2012). At the heart of SGA is the use of a compressed data structure called the FM-index, which is used to model the read sequence overlap graph of all the samples. Recently, work has been carried out to investigate building these structures using reads from multiple samples to represent all of the haplotypes present in the samples (Simpson, 2012).

An alternative approach is to first create individual whole-genome *de novo* assemblies for each sample and then subsequently carry out whole-genome alignments of the pre-assembled sequences. Several algorithms have been proposed for creating whole-genome alignments taking into account substitutions, insertions, deletions, and larger structural rearrangements. One such implementation of this approach is the combined Progressive Cactus and Hierarchical Alignment (HAL) graph pipeline (Paten et al., 2011). HAL is a graph-based hierarchical alignment format for storing multiple genome alignments arranged phylogenetically with the corresponding ancestral sequence reconstructions as internal nodes (Hickey et al., 2013).

The Mouse Genomes Project (http://www.sanger.ac.uk/resources/mouse/genomes/) has made a substantial contribution toward our understanding of structural variation diversity in mouse genomes and in their correlation to phenotypic variation. However, as explained in this review, there are ongoing challenges in computational detection of SVs with complex molecular architecture. Improved sequencing technologies with longer read lengths, along with the completion of *de novo* assemblies of mouse genomes, will be crucial in the identification of the remaining structural variants. *De novo* assembly also avoids reference bias in ascertainment of SVs (Sousa and Hey, 2013). Using longer fragments in sequencing library construction also aids in *de novo* assembly and SV detection in genomic regions that are “inaccessible” to short-read mapping due to their repetitive nature.

## Author contributions

All authors read and approved the final manuscript. Thomas M. Keane and Binnaz Yalcin wrote the paper.

### Conflict of interest statement

The authors declare that the research was conducted in the absence of any commercial or financial relationships that could be construed as a potential conflict of interest.
